# Intermittent Hypoxia Conditioning: A Potential Multi-Organ Protective Therapeutic Strategy

**DOI:** 10.7150/ijms.86622

**Published:** 2023-09-18

**Authors:** Qihan Zhang, Wenbo Zhao, Sijie Li, Yuchuan Ding, Yuan Wang, Xunming Ji

**Affiliations:** 1 Department of Neurology, Xuanwu Hospital, Capital Medical University, Beijing, China.; 2 Beijing Key Laboratory of Hypoxic Conditioning Translational Medicine, Xuanwu Hospital, Capital Medical University, Beijing, China.; 3 Emergency Department, Xuanwu Hospital, Capital Medical University, Beijing, China.; 4 Department of Neurosurgery, Wayne State University School of Medicine, Detroit, Michigan, USA.; 5 Department of Neurosurgery, Xuanwu Hospital, Capital Medical University, Beijing, China.

**Keywords:** Intermittent hypoxia, hypoxia conditioning, organ protection, clinical application.

## Abstract

Severe hypoxia can induce a range of systemic disorders; however, surprising resilience can be obtained through sublethal adaptation to hypoxia, a process termed as hypoxic conditioning. A particular form of this strategy, known as intermittent hypoxia conditioning hormesis, alternates exposure to hypoxic and normoxic conditions, facilitating adaptation to reduced oxygen availability. This technique, originally employed in sports and high-altitude medicine, has shown promise in multiple pathologies when applied with calibrated mild to moderate hypoxia and appropriate hypoxic cycles.

Recent studies have extensively investigated the protective role of intermittent hypoxia conditioning and its underlying mechanisms using animal models, demonstrating its potential in organ protection. This involves a range of processes such as reduction of oxidative stress, inflammation, and apoptosis, along with enhancement of hypoxic gene expression, among others.

Given that intermittent hypoxia conditioning fosters beneficial physiological responses across multiple organs and systems, this review presents a comprehensive analysis of existing studies on intermittent hypoxia and its potential advantages in various organs. It aims to draw attention to the possibility of clinically applying intermittent hypoxia conditioning as a multi-organ protective strategy. This review comprehensively discusses the protective effects of intermittent hypoxia across multiple systems, outlines potential procedures for implementing intermittent hypoxia, and provides a brief overview of the potential protective mechanisms of intermittent hypoxia.

## Introduction

Hormesis is defined by a biphasic dose-response relationship where low-dose exposures elicit stimulatory effects, while high-dose exposures produce inhibitory outcomes.^1^ This adaptive response serves to facilitate acclimation, thereby enhancing an organism's resilience, which can be beneficial. One such instance is the physiological hormetic responses induced by low-level exposure to hypoxia, enhancing the performance of the organism.^2^

Preconditioning and postconditioning are specific manifestations of this adaptive response induced by hormesis. Hypoxia preconditioning involves brief exposure to potentially harmful stimuli, fostering tissue and organ resistance or tolerance to subsequent injury. In contrast, hypoxia postconditioning refers to hypoxic exposure immediately after, or some time following, injury to mitigate damage and facilitate recovery.^3^, ^4^

The concept of hypoxia conditioning first emerged among Soviet pilots seeking effective methods to hasten high-altitude acclimatization.^5^ When applied intermittently, requiring periods of reoxygenation between hypoxic episodes, the protocol is termed intermittent hypoxia conditioning (IHC). Various protocols have shown intermittent hypoxia to have both beneficial and detrimental effects.^6^

Intermittent hypoxia, characterized by cyclic exposure to hypoxic conditions interspersed with short-term normoxia, is a major component of obstructive sleep apnea syndrome (OSAS), a recognized cardiovascular risk factor. OSAS may induce deleterious systemic consequences through a series of pathophysiological cascades, including promoting a hypercoagulable state.^7^, ^8^ However, paradoxically, numerous studies have demonstrated that small cycles of hypoxic exposure yield beneficial effects across multiple physiological systems, leading to increased interest in its application for disease prevention and treatment over the past decades.^9^,^10^ This review will primarily focus on the favorable physiological responses of intermittent hypoxia observed in clinical studies, provide an overview of methods and protocols for implementing intermittent hypoxia, and offer a brief description of its underlying mechanisms.

## Methods

We conducted a comprehensive literature search without any restrictions on the publication date to collect relevant reports. This research was carried out on PubMed employing the following keywords: “hypoxia conditioning”, “hypoxic training”, “hypoxic adaptation”, “intermittent hypoxia”, “interval hypoxia”, “repetitive hypoxia”, “intermittent altitude exposure”, and “intermittent hypoxia-hyperoxia”. This review incorporated pertinent clinical studies, animal research, and previous reviews, along with the references contained within these articles. We considered articles published in the English language.

## Approaches to Implementing Intermittent Hypoxia

Several methodologies exist to establish physical hypoxia, referring to direct exposure to a hypoxic environment. In animal models, physical hypoxia can be achieved by confining animals in sealed containers, but this approach is unfeasible for humans.^11^

Naturally occurring hypobaric hypoxia can be experienced through high-altitude exposure, and intermittent altitude exposure can result in intermittent hypoxia.^12^ The advantageous effects of altitude hypoxia were initially noted in high-altitude dwellers and subsequently tested in athletes for enhancing exercise performance and in mountaineers to mitigate acute mountain sickness (AMS). Nevertheless, high-altitude exposure necessitates specialized medical and travel arrangements, often hindered by mountainous terrain and climate conditions, leading to high financial costs and numerous confounding factors.^13^ Hypoxic chambers, designed for hypoxic training, provide practical means to simulate hypoxic environments, whether hypobaric or normobaric. Simulated altitude chambers were extensively utilized for athlete training several decades ago and proved to be a safe modality for disease prevention and treatment, yielding numerous protective results. However, due to the high costs and inconvenience, they gradually fell out of favor.^13^ This shift led us to focus on more practical hypoxia-inducing devices called hypoxicators, initially developed by the former Soviet Union. These devices work by producing hypoxic air in a normobaric environment for inhalation.^14^ Hypoxicators can separate oxygen and nitrogen to create hypoxic gas mixtures with desired oxygen composition, which are then safely delivered to patients through an open circuit device with a face mask.^15^ This approach has become the standard and most practical method of hypoxia delivery. Many innovative, feasible, and exciting instruments, including miniaturized ones for home use, are currently under exploration.^14^

While reduced oxygen concentration is the optimal approach to induce hypoxia, chemically induced hypoxia is also possible in strict laboratory settings. This can be achieved by administering hypoxia-mimicking agents or sedative drugs, cobalt chloride and deferoxamine are the most frequently used hypoxic mimetic agents of which.^16^, ^17^ Since there are many side effects of chemicals to human, we consider that performing chemically induced hypoxia is limited in human. Moreover, in animal models, intermittent hypoxia can be induced by administering hypoxia-preconditioned cells that were cultured in a hypoxic incubator.^18^

Since its introduction, hypoxic training has gained increasing attention in the field of sports science and is widely used to enhance individual's performance level. There are several different regimens for hypoxic training at present, the original representation of which is “live high-train high” model, that is to live and train at natural or simulated altitude conditions. To avoid decreased training intensity because of reduced oxygen uptake and detrimental effects of chronic hypoxia, the “live high-train low” (LHTL) model was developed.^19^ It is charactered by living at natural or simulated altitude conditions but training at or near sea level. LHTL has been considered as a relatively effective hypoxic model to improve athletic performance at sea level. Nowadays, with the development and widespread availability of hypoxic-inducing devices, there is growing body of research recommending the “live low-train high” (LLTH) (living at or near sea level and training at natural or simulated altitude conditions) approach as effective and efficient training modality for athletes, as well as nonpharmacological prevention and treatment method for patients with various diseases or healthy adults.^20^ Various forms of exercise performed under short-term hypoxic conditions are collectively referred to as LLTH, there are further subdivisions into different approaches within this model: intermittent or prolonged hypoxic training, repeated sprints, and resistance training.^21^ A combination of different hypoxic modalities represents a sounded approach, when athletes perform both hypoxic and normoxic training in their program while “living high”, this approach is termed “live high-train low and high”.^22^ Although these hypoxic training and exposure methods differ, they all share the same goal: to improve athletic performance at sea level.

## Protocols and Parameters of Intermittent Hypoxia

While the partial pressure of oxygen is typically considered the primary physiological stimulus for hypoxia adaptation, recent evidence suggests that hypobaric hypoxia (HH) and normobaric hypoxia (NH) may not be entirely equivalent.^13^, ^23^ Aside from oxygen concentration, barometric pressure may independently affect many physiological responses.^24^ In a study designed to compare changes of athletes under NH and HH conditions with matched inspired oxygen partial pressure, researchers found that subjects who underwent HH exposure tend to have greater performance enhancements and hematological changes, however, it is possible that these results may have been influenced by the higher hypoxic stimuli in the HH exposure group.^25^ It was then reported that increase of maximal oxygen consumption, performance enhancement and hemoglobin mass were similar between the two conditions in crossover studies.^26^, ^27^ Nonetheless, safety considerations should not be neglected. Intermittent HH could induce unfavorable biochemical alterations, including reduced oxidation resistance and heightened lipid peroxidation, potentially impairing vascular endothelial function and vascular hemodynamics. Therefore, appropriate hypoxic modes should be chosen based on the specific practical application.^28^

Traditional IHC is defined by cyclic alternation between hypoxia and normoxia. A recent adaptation substitutes atmospheric normoxia with a hyperoxic mixture, with the inspired oxygen fraction (FiO_2_) ranging from approximately 30% to 40%, this protocol is known as intermittent hypoxia-hyperoxia. In human studies comparing intermittent hypoxia-hyperoxia and hypoxia-normoxia, evidence suggests that these two approaches have comparable effects in improving metabolic parameters and promoting angiogenesis.^29^, ^30^ But animal research found that intermittent hypoxia-hyperoxia has greater protective preventive effect than intermittent hypoxia-normoxia does.^31^ Further studies are warranted to better understand the biological consequences and possible risks in replacing normoxia with the hyperoxia in intermittent hypoxia conditioning.

Several parameters should be considered in defining the IHC protocol, including the severity of hypoxia, the duration of hypoxic intervention, the number of hypoxic episodes, and the frequency of hypoxic stimuli, collectively referred to as the hypoxic “dose”. The optimal oxygen concentration to induce beneficial outcomes is yet to be established and is usually tailored to the objectives of the study. Some research uses a target peripheral oxygen saturation to define hypoxic severity instead of specifying the inhaled oxygen concentration.^32^ The duration of each hypoxic cycle can vary from seconds to hours, and the number and frequency of IHC episodes can also range widely, from a single hypoxia exposure to several daily repetitions over days or weeks.^6^, ^33^, ^34^ Depending on the experimental paradigm, biological responses to IHC could be adaptive or maladaptive. While no universally accepted optimal protocol for IHC exists, emerging evidence suggests that IHC protocols characterized by mild to moderate hypoxia (9-16% inspired oxygen), short durations (3-10 min), and 3-15 cycles/day generally yield beneficial outcomes both in models of disease and human subjects.^6^, ^34^

## Clinical Applications of Intermittent Hypoxia Conditioning

**Figure 1:**
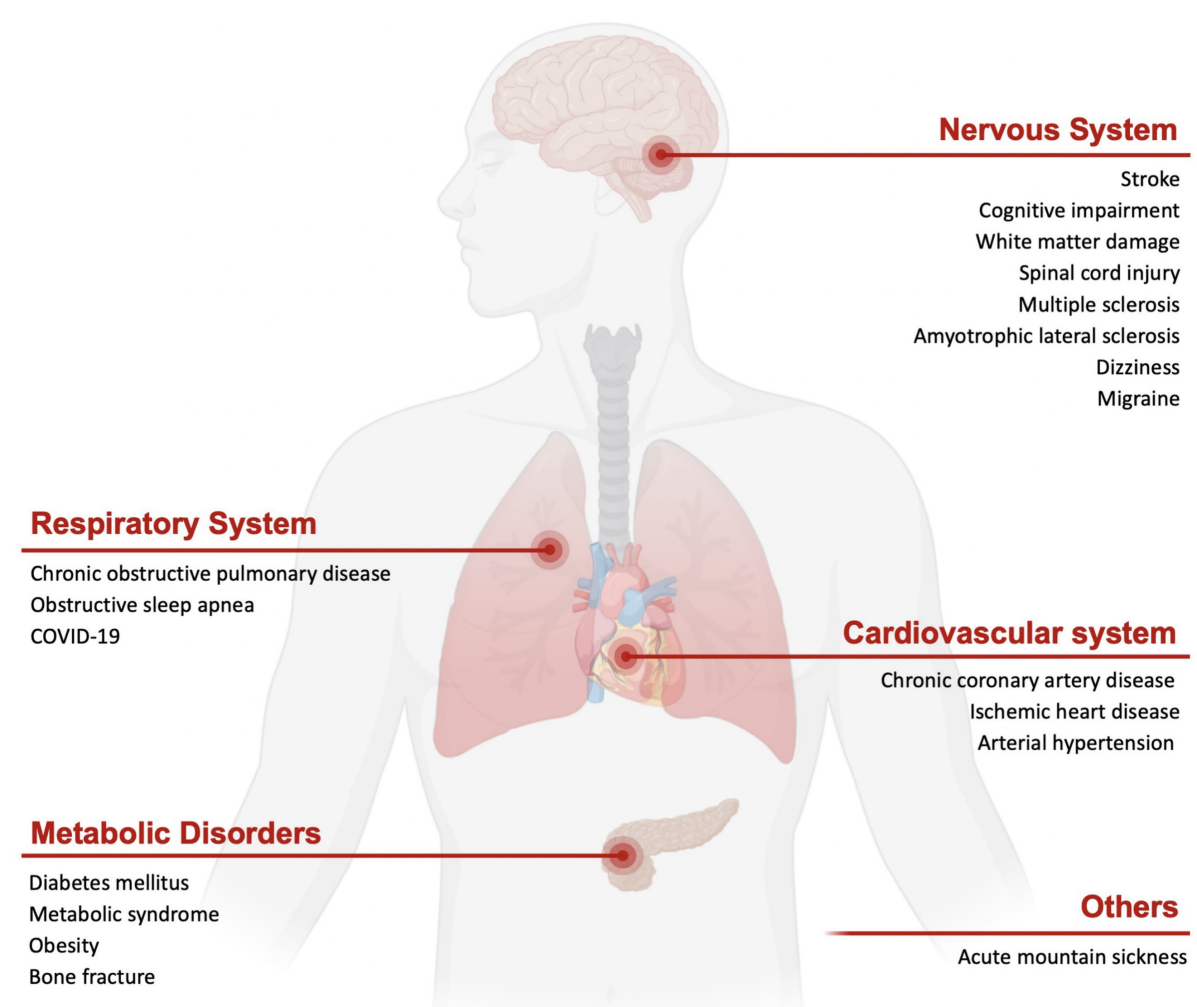
** Schematic of the potential efficacy of intermittent hypoxia conditioning in different systems of disease.** IHC impacts the entire body, such as through neural, respiratory, and cardiac and metabolic protection.

Intermittent hypoxia has emerged as a potential nonpharmacologic strategy that harnesses humans' inherent adaptive defenses in both healthy individuals and patients. The direct and cross-advantages of IHC are applied in a range of diseases. This section largely delves into the practical implementation of IHC for systemic diseases in humans (Refer to Figure 1).

### Nervous System

Stroke, a leading global cause of mortality and disability, has been the focus of numerous studies aiming to lessen the damage and enhance rehabilitation.^35^, ^36^ Several studies since the 1990s have reported the use of IHC in neuroprotection to diminish infarct size, alleviate ischemia-reperfusion injury, and bolster neurological recovery in stroke models.^37^ Clinical research suggests that a history of ipsilateral transient ischemic attacks, the temporary episodes of brain ischemia, can generate ischemic and hypoxic preconditioning.^38^ This may induce ischemic tolerance leading to milder functional deficits at stroke onset and a better prognosis. Poststroke intermittent hypoxic exposure (FiO_2_=20.9%~15%) combined with intermittent hypoxic training (five cycles of 2-min hypoxia at FiO_2_=15% interspaced with 2-min normoxia intervals) has aided in the secondary prevention and functional recovery of stroke patients.^39^ Furthermore, neural stem cells preconditioned with 5% hypoxia for 24 hours can enhance the efficiency of stem cell therapy for intracerebral hemorrhage in mice.^40^

A pilot study indicated that intermittent hypoxia-hyperoxia training (four cycles of 5-min hypoxia at FiO_2_=12% separated by 3-min hyperoxia at FiO_2_=33%, five sessions per week for three weeks) enhances cognitive function in elderly patients with mild cognitive impairment, a precursor to Alzheimer's disease (AD), with a long-lasting therapeutic effect.^41^ It significantly reduces circulating AD marker levels, including amyloid precursor proteins and amyloid beta (Aβ).^41^, ^42^ An intermittent hypoxia protocol of four cycles of 10-min hypoxia and 5-min normoxia for 18 sessions combined with physical exercise can boost exercise performance and augment the positive effects on cognitive function in elderly individuals.^43^ A recent study investigating the effects of IHC on cerebral white matter damage concluded that intermittent hypoxia (5-min hypoxia at FiO_2_=11~12% and 3-min normoxic intervals, eight sessions for 60min/day for 10 days) might reduce sustained normobaric hypoxia-induced white matter damage.^44^ It was evidenced that IHC for 14 days or 28 days reliefs the progression of AD by alleviating memory deficits, inhibiting Aβ accumulation and reducing inflammation of brain in mice model, which offers new insights to AD therapy.^45^

Spinal cord injury (SCI), a severe traumatic nervous injury, often results in degrees of respiratory insufficiency and significant limb and trunk dysfunction. In these patients, single-session or repeated sessions of IHC can potentially enhance respiratory neuroplasticity after the decline of respiratory function associated with neurological injury, likely by increasing maximal inspiratory pressure and training the respiratory system.^15^, ^46^ Moreover, daily exposure (5consecutive days) of intermittent hypoxia (1.5 minutes hypoxia of FiO_2_=9~10% with 1-min normoxic intervals for 15 episodes) combined with conventional practices has been shown to expedite motor rehabilitation, including walking performance and hand function in patients with chronic incomplete SCI.^47^, ^48^ In rat models, transplantation of hypoxia-preconditioned bone mesenchymal stem cells alleviated SCI and facilitated functional recovery by improving cell survival and migration rates.^17^ As fatigue is one the most frequent symptoms of individuals with multiple sclerosis (MS), a pilot study indicated that 14 days of hypoxic training (with the oxygen concentration of 15% to 16.4%, 60 min per day) could positively influence walking endurance in MS patients.^49^ An IHC protocol of 1-min hypoxia at FiO_2_=10% interspersed by 2-min normoxia for 15 episodes is a promising and safe method to increase collective inspiratory muscle activity in amyotrophic lateral sclerosis patients, further clinical trials of repetitive IHC are warranted to confirm these respiratory functional benefits.^50^

Normobaric IHC (5 cycles of hypoxia at FiO_2_=10% for 5 min followed by normoxia for 5 min) for four weeks significantly improved symptoms and reduced the frequency of dizziness episodes in patients suffering from dizziness.^51^ The effect of IHC in minimizing dizziness might be due to the improvement of vasomotor functionality and the balance of cerebral neurotransmitter release. A similar eight-week IHC protocol was found to be effective in alleviating migraines, with therapeutic effects persisting up to three months post-intervention.^52^

Animal studies suggest that intermittent hypoxia could protect rodent models from stress-related and hypoxia-induced depression and anxiety,^53^, ^54^ which needs to be further investigated before possibly applying to humans. In addition, IHC was found to be effective in suppressing hypoxia-induced neuronal dysfunction by inhibiting inflammation and promoting neural stem cell proliferation in mice, which provides a novel concept for the development of the treatment of hypoxia-related brain injury.^55^

### Cardiovascular System

In myocardial infarction (MI) model, hypoxic preconditioned stem cell-derived extracellular vehicles (EVs) show more improvement in cardiac repair than that cultured in normoxic conditions.^56^ IHC could typically reduce infarct size, minimize arrhythmias, and inhibit atherosclerosis development in animal models by improving myocardial vascularity and coronary blood flow in MI and ischemic/reperfusion models.^57^-^59^ Some of these effects have been verified clinically in patients. For those with coronary artery disease, 15 sessions of intermittent hypoxia-hyperoxia conditioning (FiO_2_=10% and 30%, respectively, for 5 to 7 cycles lasting 4 to 6 minutes in each session) are associated with enhanced exercise tolerance, improved risk factor profile, and better quality of life.^60^ In patients undergoing coronary artery bypass surgery, four daily intermittent hypoxia-hyperoxia prior the operation has been proved to improve the injury during operation, as indicated by decreased serum concentration of troponin I and lactate after surgery than those in the control group receiving normoxia.^61^ A preliminary study suggested that 14 sessions of intermittent hypobaric hypoxia (equivalent to a simulated altitude of 4,200m) could markedly enhance myocardial perfusion, as evaluated by perfusion imaging, in patients with severe stable coronary artery disease who have undergone coronary bypass surgery 6 months ago.^62^ In elderly patients with stable angina, it was discovered that 15 sessions of intermittent hypoxia-hyperoxia can safely reduce angina's clinical symptoms and the daily duration of myocardial ischemia.^63^ Endurance training in intermittent hypoxic conditions (70 min hypoxia exposure at FiO_2_=16.8%, 5 times a week for 21 days) could increase exercise tolerance and left ventricular ejection fraction, the primary parameters determining prognosis, in patients with MI.^64^

Clinical trials have confirmed that intermittent hypoxia has antihypertensive effects in patients with arterial hypertension.^65^, ^66^ This may be associated with increased endothelial nitric oxide (NO), hypoxia-inducible factor 1 (HIF-1), and angiogenic growth factors, reduced sympathetic activity, and improved water and salt metabolism.^6^, ^66^ Furthermore, in hypertensive patients with obstructive sleep apnea, 15 sessions of mild intermittent hypoxia exposure when awake, coupled with nightly continuous positive airway pressure (CPAP), reduced their blood pressure.^10^ This was not observed in patients solely following CPAP treatment.

### Respiratory System

Evidence underscores the significant role of cardiovascular autonomic dysfunction in patients with chronic obstructive pulmonary disease (COPD). Mild intermittent hypoxia for 15 sessions might serve as a potential therapeutic avenue to enhance autonomic cardiovascular and respiratory control.^67^ This is achieved by augmenting the hypercapnic ventilatory response and restoring baroreflex sensitivity to normal levels, thereby boosting their forced expiratory volume in one second and forced vital capacity without adverse side effects in COPD patients.^67^, ^68^

Mild intermittent hypoxia triggers forms of respiratory plasticity that is the adaptive response of systems to stimuli. This includes increasing the stability of the upper airway, reducing the therapeutic CPAP necessary for treating sleep apnea, and positively affecting comorbidities associated with OSAS.^69^ The progressive amplification of the hypoxic ventilatory response and long-term facilitation of ventilation in OSAS patients, induced by intermittent hypoxia, position it as an effective adjunct therapy for sleep apnea.^68^

Additionally, IHC might serve as a potential strategy for the rehabilitation of 2019 coronavirus disease (COVID-19) patients by mitigating their severe dyspnea through enhanced blood oxygen delivery and tissue oxygenation response.^70^ But randomized controlled trial concluded that the combination of moderate-intensity training with moderate hypoxia promoted similar benefits in cardiorespiratory fitness with that of normoxia in patients recovered form COVID-19.^71^ As the symptoms and pathomechanisms of COVID-19 are not limited to the respiratory system, appropriate timing and protocols to apply IHC for rehabilitation should be considered under the premise of safety.

### Hematological System

It has been reported that moderate intermittent hypoxia training or exposure can enhance both aerobic exercise performance and anaerobic capacity in athletes.^21^, ^72^, ^73^ However, as we know, it is not appropriate to talk about effects without considering the hypoxic “dose”. Due to methodological differences, the efficacy of intermittent hypoxia in enhancing exercise performance is controversial, several studies have also concluded that intermittent hypoxia exposure does not show positive effect in improving athletes' performance.^74^, ^75^ While multiple studies have highlighted the therapeutic benefits of coupling exercise with hypoxia as an effective non-pharmacological therapy in systemic diseases.^76^, ^77^

The potential mechanism by which hypoxic training can enhance exercise performance is also controversial. Although it is widely believed that altitude training may lead to beneficial changes of hematology, it may not be the main, or the only, factor related to performance enhancement. In some studies, hematological profile alterations are evident in improved erythropoiesis and blood oxygen transport capacity, as indicated by significant increases in hematocrit, red blood cell count, reticulocyte count, and hemoglobin concentration in subjects exposed to different intermittent hypoxia protocols.^78^, ^79^ Moreover, erythrocyte P50, a representation of hemoglobin-oxygen affinity, was significantly increased after 10-day intermittent hypoxia exposure (5-min moderate hypoxia with 3-min normoxic intervals for eight sessions) compared to the that in the control group, suggesting that IHC enhances the oxygen release capacity of erythrocytes.^44^ Conversely, some studies did not observe changes in hematological indices following four-week intermittent hypoxia training in highly trained athletes, which may be explained by insufficient hypoxia exposure time to accelerate erythropoietic response.^80^, ^81^ These discrepancies might be attributed to the varied hypoxic protocols employed or high individual variability in acclimatization to hypoxia.^82^

### Metabolic Disorders

Past research has shown that a single session of IHC (6-min hypoxia at FiO_2_= 13% alternated by 6-min normoxia for 5 cycles) can decrease blood glucose levels in individuals with type 2 diabetes (T2D) without autonomic complications.^83^ This effect is achieved by reestablishing the balance of primary cardiorespiratory reflexes, leading to a sustained reduction in blood glucose. The similar IHC protocol on type 1 diabetes also yielded positive results.^84^ Hypoxic exposure during exercise, in comparison to normoxic conditions, may enhance glucose uptake and insulin sensitivity in T2D patients, providing further benefits.^85^ In patients diagnosed with metabolic syndrome, characterized by impaired glucose metabolism and insulin resistance, fifteen sessions of intermittent hypoxic-hyperoxic exposures appeared to improve the lipid profile, reduce systemic inflammation, and adjust cardiovascular and metabolic profiles.^86^, ^87^

In obese individuals, passive hypoxic exposure triggers beneficial cardiovascular and respiratory adaptations despite no alternation in anthropometric data.^88^ Combining exercise with hypoxic exposure is considered a promising supplementary approach for obesity management.^89^ Additionally, a 1-week exposure to high altitude (2,650m) without additional exercise showed weight reduction in obesity subjects.^90^ The suppression of appetite-related hormones, such as leptin, in hypoxic conditions contributes to a reduction in appetite and dietary energy intake, subsequently leading to weight loss. Moreover, as compared to training under normoxic conditions, hypoxic training can enhance fatty acid utilization, reduce body fat percentage, and improve metabolism.^91^

Intermittent hypoxia aids bone fracture healing by inhibiting osteogenic differentiation and enhancing osteoclast function in rats. This mechanism is associated with the enhancement of HIF-1 expression-linked signaling pathways.^92^ Further clinical studies are required to evaluate the impacts of intermittent hypoxia on bone metabolism.

### Other Applications

Acute hypoxia injury can occur during sudden exposure to high altitudes. However, repeated short-term hypoxic preconditioning has the potential to mitigate these effects. A randomized controlled study found that short-term IHC (1 h/day at FiO_2_=12.6% for 7 consecutive days) facilitated high-altitude acclimatization and reduced the severity of AMS, as evaluated by the Lake Louise score.^93^ In addition to decreasing the severity of AMS, four hours hypoxia exposure at FiO_2_=12% daily for 4 days has the potential to reduce its incidence by reducing high-altitude-induced dyslipidemia and inflammation.^94^ The impairment in decision-making, which can occur in mountaineers and potentially lead to dangerous consequences, can be minimized via 7-day intermittent hypoxia preconditioning.^95^

## Potential Mechanisms Activated by Intermittent Hypoxia

**Figure 2:**
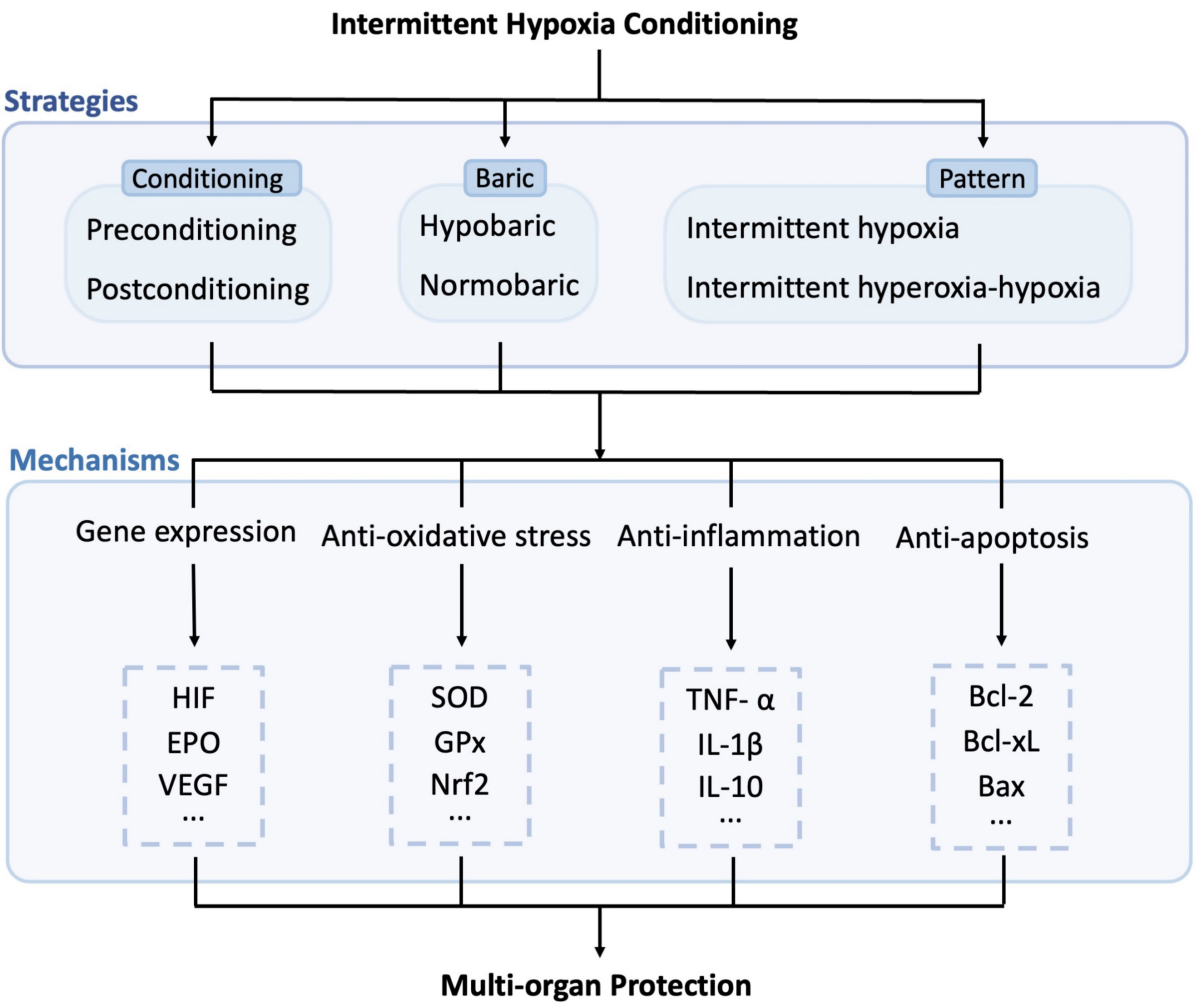
** Potential molecular mechanisms induced by intermittent hypoxia conditioning.** IHC exerts multi-organ protection effects through multiple pathways, including enhancing gene expression, reducing oxidative stress, inflammation, and apoptosis. HIF: hypoxia-inducible factor; EPO: erythropoietin; VEGF: vascular endothelial growth factor; SOD: superoxide dismutase; GPx: glutathione peroxidase; Nrf2: nuclear factor erythroid-2 related factor 2; TNF-α: tumor necrosis factor, IL-1β: interleukin 1 β; IL-10: interleukin 10; Bcl-2: B-cell lymphoma-2; Bcl-xL: B-cell lymphoma-extra-large; Bax: Bcl-2-associated X.

Short-term exposure to mild hypoxia protects cells, tissues, or organs from more severe hypoxia and ischemic insults. Understanding the various mechanisms of action for IHC in specific applications is valuable for establishing the most effective IHC protocols for each condition. Since several comprehensive reviews on the complex array of mechanisms of IHC from systemic physiological reactions to genomic regulation exist,^4^, ^57^, ^96^, ^97^ this section provides a succinct summary (Refer to Figure 2).

Intermittent hypoxia-normoxia triggers two potent transcriptional mediators, HIF-1 and nuclear factor erythroid-2 related factor 2 (Nrf2). These mediators promote the expression of subsequent cytoprotective proteins. HIF-1, a heterodimeric, helix-loop-helix protein, consists of two subunits that join in the nucleus to form a functional complex under hypoxic conditions. This complex activates the transcription of target genes, such as erythropoietin (EPO) and vascular endothelial growth factor (VEGF).^98^ EPO is known as an effective agent against ischemic injury, particularly in cardiac, renal, and neural protection, through activating several protective signaling pathways.^99^ VEGF also plays a role in angiogenesis and neurotrophic processes by increasing vascular density and blood flow.^3^, ^100^ As such, cerebral ischemia injury could be significantly reduced following hypoxic conditioning due to the increased expression of HIF-1 and its target genes.

Nitric oxide has a cytoprotective role due to its vasodilatory effects and regulation of mitochondrial function. The inducible nitric oxide synthase gene, along with two other isoforms, is promoted by HIF-1 in response to intermittent hypoxia, enhancing NO synthesis capacity. NO can reduce infarct size by increasing coronary and cerebral blood flow around the ischemia risk area. Moreover, it exerts antioxidative stress effects in various conditions.^97^ Intermittent hypoxia exposure can activate antioxidant enzymes, including superoxide dismutase and glutathione peroxidase in brain injury model.^101^ Aside from regulating antioxidant enzymes, IHC also reduces cerebral injury through antioxidant stress by activating Nrf2, a master redox regulator, the upregulation of which could protect the brain from oxidative stress damage.^102^ Reactive oxygen species (ROS), one of the key triggering mechanisms for adaptive responses to IHC, is generated in the initial period of reoxygenation. ROS can trigger hypoxia-induced transcription and initiate expression of certain genes with the effect of increasing antioxidant capacity to promote defense against cellular stress.^29^

Inflammation is a double-edged sword, the appropriate duration and degree can help maintain homeostasis, but an excessive inflammatory response is insalubrious. Evidence suggests that inflammatory factors are implicated in IHC-mediated protection. Hypoxic preconditioning could inhibit microglial polarization and subsequent inflammatory responses.^103^ IHC can potentially mitigate microglia-induced brain injury by suppressing proinflammatory cytokines (tumor necrosis factor-α, interleukin 1 β, and interleukin 6) and upregulating anti-inflammatory cytokines (interleukin 10).^55^, ^104^ The anti-inflammatory effects of IHC are also associated with affecting the chemotaxis of peripheral immune cells such as suppressing early leukocyte infiltration.^105^ In addition, transcription factors, such as anti-apoptotic B-cell lymphoma-2 (Bcl-2) and B-cell lymphoma-extra-large, pro-apoptotic Bcl-2-associated X, involved in the regulation of apoptosis and autophagy activate the gene expression of related proteins following acute intermittent hypoxia exposure.^106^-^108^

Heat shock protein 70, regulated by HIF-1, is believed to have cytoprotective roles in the processes of ischemic apoptosis and autophagy. It can act as a molecular chaperone to repair or remove proteins denatured by stresses, thereby increasing resistance to ischemia/reperfusion injury after intermittent hypoxia exposure.^109^

Furthermore, ATP-dependent potassium channels may be involved in enhanced tolerance to ischemic injury after intermittent hypoxia adaptation, as their activation inhibits calcium overloading induced by ischemia/reperfusion.^110^ The protective mechanisms of intermittent hypoxia that induce or maintain hypoxia tolerance are complex and interact with each other. Identifying a method to achieve long-term protection remains a significant challenge.

## Perspective and Prospective

Decades of systemic research have highlighted the profound and often unexpected effects of intermittent hypoxic techniques. The crux of our review is that IHC yields a range of physiological benefits and presents a reliable nonpharmacological approach. Thus, we advocate for the appropriate application of intermittent hypoxia across diverse clinical conditions, given its plethora of evident benefits and minimal risks. However, the optimal IHC protocol is not a one-size-fits-all solution, varying according to specific clinical conditions. There is significant heterogeneity due to individual factors such as comorbidities, medications, patient status, and other variables, requiring tailored conditioning protocols to induce optimal stimulation. With the impact of IHC differing from disease to disease and individual to individual, comprehensive evaluations of the risks versus benefits of IHC are necessary. This will facilitate the development of a series of standardized, practical IHC guidelines tailored to individual patient needs and specific clinical contexts.

As we look to the future, exploring the combination of IHC with conventional treatments such as medications and physical exercise is crucial. These combined strategies have the potential to potentiate or extend the effect of IHC, creating a more holistic and efficacious treatment approach. The interdisciplinary nature of this approach could lead to innovative breakthroughs in treatment protocols. In addition, further investigation into the molecular mechanisms underlying the effects of IHC could lead to the refinement of IHC protocols and the development of novel therapeutic strategies. Understanding these mechanisms on a deeper level would provide a scientific foundation for customizing IHC protocols to suit individual patient needs more accurately. Finally, large-scale, randomized controlled trials are needed to confirm the effectiveness and safety of IHC across different diseases and populations. Such studies could help establish the role of IHC in mainstream clinical practice and provide robust evidence to support its adoption on a wider scale.

In conclusion, while IHC is a promising treatment modality with numerous potential benefits, its implementation should be evidence-based, patient-specific, and incorporated in a manner that complements existing treatments. As we continue to research and understand the nuances of IHC, we believe it holds significant potential for improving patient outcomes across a broad spectrum of clinical conditions.

## Abbreviations

IHC: intermittent hypoxia conditioning
OSAS: obstructive sleep apnea syndrome
AMS: acute mountain sickness
LHTL: live high-train low
LLTH: live low-train high
HH: hypobaric hypoxia
NH: normobaric hypoxia
FiO_2_: inspired oxygen fraction
AD: Alzheimer's disease
Aβ: amyloid beta
SCI: spinal cord injury
MS: multiple sclerosis
MI: myocardial infarction
EVs: extracellular vehicles
NO: nitric oxide
HIF-1: hypoxia-inducible factor 1
CPAP: continuous positive airway pressure
COPD: chronic obstructive pulmonary disease
COVID-19: 2019 coronavirus disease
T2D: type 2 diabetes
Nrf2: nuclear factor erythroid-2 related factor 2
EPO: erythropoietin
VEGF: vascular endothelial growth factor
ROS: reactive oxygen species
Bcl-2: B-cell lymphoma-2
